# Mississippi Valley Medical Association

**Published:** 1888-11

**Authors:** 


### MISSISSIPPI VALLEY MEDICAL ASSOCIATION.

The annual meeting was held at St. Louis, September 25, 26, 27, twelve States being represented.

Mayor DAVID R. FRANCIS welcomed the visitors on behalf of the city.

From the standpoint of morality, society, and religion, the doctors of the country went far toward molding the public mind; in the last of these three they were, perhaps, not always sound, and were sometimes too materialistic and unorthodox, but were always sincere.

The Mayor hoped and believed that the visitors would be so well entertained while in the city that they would not only prolong their stay, but would return again, and often. The conclusion of his remarks was a reiteration of his more than hearty welcome to the city on the part of her people.

Professor LOUIS CH. BOISLINIERE, welcomed them on behalf of the medical profession of St. Louis, and said:

In the near future our glorious Western States will cease to be tributary to the East, In the Mississippi Yalley intellectual centres are even now beginning to assert themselves. St. Louis, Chicago, Louisville, Cincinnati are the rising stars in the firmament of the future, and already form a brilliant constellation. They are justly proud of their medical colleges, universities, hospital and clinical facilities, so great, that no student needs go East for instruction. We are also having our great men, and mean to keep them. The great West has heretofore been the nursery of men of the highest eminence, and has given to the East, Gross, Marion Sims, Flint, Sayre, Parvin, Bartholow, Meigs, Emmet, Thomas, and other brilliant luminaries. Our great men of the future will no longer leave us in search of higher honors. The growing West will be a field vast enough for their noblest ambition.

The President, Professor Dudley S. Reynolds, presided.

The first paper was by Professor C. G. Comegys, of Cincinnati, in which "Yellow Fever" was discussed from a clinical standpoint, giving a typical case of the disease, which was followed by general discussion.

In closing the discussion, Professor COMEGYS said: I certainly agree with what has been said on the non-contagious nature of the disease. It is the clothing that spreads the disease. If a man be taken from a yellow-fever district, stripped of all his own clothing and given a new outfit, there is no danger of catching yellow fever from him.

TUESDAY, SEPTEMBER 25.

This was followed by a discussion of infant-feeding.

Dr. LARRABEE: In connection with this subject, there is another thing which should be considered along with it, the deterioration at the present day of our women on account of over-stimulation so common now. Up to within the last few years the question of infant-feeding was hardly considered, because of the fact that mothers were able to nurse their own children. This is not the case now, and the question of infant-feeding has taken a very important place in medical science. Of course, no food has as yet been found to take the place of the natural supply, and I think I can safely say that none such will be found. As the nearest approach to this natural food is the good old-fashioned family cow, pure milk, fresh from the cow, is the best.

The best of artificial foods are the hydrocarbons. A point in artificial feeding, and an important one, is that in almost every instance of artificially-fed babes, they are overfed. It is exceedingly difficult for the mother or nurse to distinguish between the cry of colic, made by a stomach distended with food, and the cry of hunger; and in the doubt in this case there is no more convenient a stopper than the bottle, and consequently that infant is continually overfed.

It ought to be known that every cry is not necessarily for food. Every infant ought to have water, and not always food, but it ought to be known, also, that the child requires a little salt now and then. The latter ingredient has done more in my hands in facilitating osmosis than all other medicines I have used. Another point, the artificially-fed child is kept always upon one diet. The mother who nurses her babe, gives it such diet as accident or inclination may decide, and the child feels the benefit or injury of such changes.

Dr. E. P. COOK, of Mendota: I have usually been so fortunately situated that when Nature has failed to supply the wants of the human offspring, my general resort has been to that which to my mind comes nearest to the mother's milk, that of the healthy cow. I only will add, that in my own observation those means that keep the milk in as pure and as nearly perfect a condition as possible with as little addition or change, by means of anything to preserve it in good condition—I mean as we ordinarily observe the sterilization of that milk that is intended to be given to the human infant—is one of the important subjects aside from the care in the selection of the healthy cow from which we obtain it. Of course, the milk of the healthy mother is best, but where it is necessary to make a substitute, let it be that of the cow. I don't know much about the various devices for keeping milk in good condition, but I am confident that if it is taken quickly and subjected to heat, placed in conditions where there is little or no chance of atmospheric infection, we have in that case the best thing as substitute for the mother's milk. I have been in the habit of immediately closing milk in fruit-jars, placing it in the ice-box and keeping it there until it is drawn upon. I know but little about those various substitutes of artificial food. I am glad to say I have had very little necessity to become familiar with them.

Dr. MAYFIELD, of St. Louis: I have adopted a method of sterilizing milk that has worked satisfactorily with me for twelve months. I took the suggestion from a milk company. The milk is sealed as soon as taken from the cow; then to sterilize this I have a water-tank. We heat the water for twenty-four hours, keeping it at the boiling point, and this water is first purified before it reaches a receiver, by running through charcoal, and from there we draw it directly into the milk, making the preparation half water and half milk, adding sugar as necessary. I have used the various preparations of food as they come from the manufacturer, and a great many of them have given considerable satisfaction, but not as much as the milk prepared as I have stated.

Dr. N. GUHMAN, of St. Louis: It seems to me that the previous speakers have forgotten that the condition of the stomach of the child has a good deal to do with the diseases of the children. If we would make more use of antiseptics with the digestants, it would be better. We have to find out what is especially applicable in each case; it is the same thing with ourselves, we don't digest the same things in the summer that we do in the winter.

Dr. BEARD, of Chicago, read a paper on "Diseases of the Middle Ear."

Dr. DICKINSON, of St. Louis, discussing the same, said: I think that this disease, as a general thing, is neglected, and cited one unfortunate case as an illustration of such neglect. No attention was paid to it until some years afterward. At the time I saw the patient, this physician had deceased. On inquiring, I learned from the mother that at times little white substances had passed, and so probably all of the ossicula had come away. There was total occlusion of the meatus, and hearing was totally destroyed, rendered so by this closure. The other ear was similarly affected, but not to the same extent. Treatment within a short time arrested the process. Dr. Beard made a remark regarding the suppurative conditions of the middle ear: that they do admit of cure but the chance is not always given. A physician told me he had six cases which terminated fatally during the winter; therefore these affections of the middle ear are not trifling in character, and therefore deserve the attention of the general, as well as the special, practitioner.

Dr. D. A. THOMPSON, Indianapolis: In regard to the catarrhal affections, the time we usually look for them and their causes is in early infancy; they then develop this tendency and are subject to the changes in temperature; then is the time for the general practitioner to look after them. Some of these suppurative affections seem not to yield to any treatment.

The PRESIDENT: The presence of discharge from the ear should awaken suspicions that should reject an applicant for life insurance. I knew a man not long since who, in apparently good health, died within two weeks from a perforating abscess into the brain.

Dr. BEARD: I don't question the incurability of some cases, and I agree that some seem not to yield to the treatment I have mentioned, but it has been my experience that they are, as a class, a very satisfactory kind of disease to deal with.

Dr. WM. DICKINSON, of St. Louis, read a paper on "Retinitis Albuminurica," and gave details of a case presenting the characteristic features of this affection.

The presence of this affection simultaneously with the pregnant state was to some extent discussed, and the urgent necessity for immediately inducing premature labor, if it occurs during the seventh month of gestation or earlier, in order to avoid blindness or death from convulsions.

Should it occur during the ninth month recovery of normal vision usually occurs after delivery; if in the eighth month, and the induction of premature delivery is not performed, only one-half of the cases recover; if at an earlier date, blindness or death frequently occurs, the danger of the supervention of these events being proportionate to the time of its invasion prior to the time of normal delivery.

The great practical lesson to be derived is never to disregard or underrate the significance of progressive failure of vision, especially when the cause is obscured.

Dr. BAILEY, of Louisville: I would ask you to widen the aspect of Bright's disease, not as simply a disease of the kidneys; the kidneys are not only involved; it is a general systemic disease, and hence this affection of the eye I would be disposed to regard rather as a part of the general disease than as a complication.

Some of these diseases are recognized more quickly in the eye than in the kidney. I believe it is constitutional always, in which there is connective-tisssue hypertrophy, resulting in hypertrophy of the left ventricle. I don't believe that a simple obstruction in the kidney would account for all the vascular changes.

I am inclined to take issue with the management of this disease as it occurs in pregnancy. I would put it on the same basis that I would a threatened convulsion due to the same cause, and I believe we can treat satisfactorily not only the disease, but by increasing the elimination from the kidneys we can save the patient from disastrous results and carry her to full term and delivery. So I would question the positiveness of the assertion that we do not wait a single hour, but remove the offending cause.

Dr. DICKINSON: I have no practical experience in this connection with pregnancy, and consequently I have to take the testimony of others; and authors on this point have been very emphatic in regard to the production of premature labor in such cases.

Professor D. R. BROWER, of Chicago, read a paper on "Exophthalmic Goitre and Its Treatment by Strophanthus."

He invited attention to the more prominent features in the clinical history and diagnosis of the disease, and then suggested its treatment by strophanthus.

The eyes, the thyroid gland, and the circulatory system are usually all changed by the morbific process in this disease. Usually the circulatory disorders are first manifested. These consist of increased frequency of the pulse, palpitation and dilatation of the arteries. The pulse, in a mild case, is about 100, but it may reach a point of much greater frequency. In the early stages the heart sounds are normal, but anæmic murmurs may soon be found, and, later, organic murmurs, that have resulted from the dilatation of the heart. The exophthalmus usually appears soon after the swelling of the thyroid, but occasionally before it, and still more rarely may precede it and the cardiac symptoms. The degree varies from a slight prominence of the eye to such protrusion that no part of the globe is covered by the lids. It is usually bilateral, not always symmetrical. It often appears earlier in one eye than in the other, and the prominence of the one may exceed that of the other. Ophthalmoscopic examination will show pulsation in the arteries of the retina.

Von Graefe first called attention to another useful sign about the eyes—the want of harmony between the movements of the eyeball and the upper lid. If the patient be directed to look downwards it will be found that the upper lid does not follow the movement of the eye, or only abnormally. The temperature of the body is elevated usually from one-half to one degree Fahr. A muscular tremor, sometimes coarse and jerky, like chorea, and sometimes fine and regular, like paralysis agitans, is present in almost every case. It is confined chiefly to the larger muscles. The head, fingers, and toes are usually exempt from this tremor. There are, also, constitutional disturbances. The gastro-intestinal area shows loss of appetite, impaired digestion, nausea, vomiting, and diarrhœa, with marked emaciation. The nutrition of the brain suffers much, as is manifest in insomia, headache, dizziness, weakness of thought and memory, irritability of temper, neuralgia. The milliampere-meter gives valuable information, as first suggested by Dr. Vigorius, by determining the condition of electrical resistance, which, in this disease, is markedly diminished.

Dr. A. A. Eshner believes that the symptoms are due to a disorder, functional in character, in the medulla oblongata, involving the cardio-inhibitory centre, the vaso-motor centre, the respiratory centre, and the other important centres located in that part. Careful consideration of the physiological functions of these centres may enable us to work out the pathogenesis of the disease.

Its treatment has been very unsatisfactory. The drugs in ordinary use have had but very little influence upon it, but Professor Brower thinks that in strophanthus we have a remedy that in some cases is of great value. He has administered a tincture of strophanthus in three cases with beneflt.

Dr. Dickinson: In this disease the vaso-motor system has lost its control, and then we have the action of the heart irregular and frequent. The writer speaks of a case in which the pulse reached 116. In a case which I recollect it was so frequent that it could not be counted. Strophanthus was not used. I had to fall back on what my ingenuity could devise. I used digitalis and the galvanic current, by which the pulse was reduced to eighty-two, and at one time to sixty-eight, after a few days.

Wednesday, September 26.

Dr. C. S. Bond, of Richmond, Indiana, read a paper on "Conditions Which Precede Serious Lesions of the Kidney." He assumed the position that long before organic kidney lesions were to be discovered there were changes in the blood as to quality and physiological elements sufficient to lead the careful practitioner to proper appreciation of the case.

No ailment complained of by patients was too trifling to deserve correct diagnosis, and seemingly slight indispositions, continued for any length of time, were to be regarded with suspicion necessary to procure for them careful inquiry—such as pains in the back and loins, susceptibility to the influences of cold beyond a normal condition, pain or disagreeable feeling in the eyes upon their exposure to light, abnormal sounds of the heart, not pronounced enough to be designated as due to organic disease, and tendencies on the part of the various organs to disturbances of function.

He dwelt upon the circumstance of an increased quantity of urea in the urine, and urged the diagnostician to inquire into this, as many times a diagnosis could be made sufficiently early to abort a commencing Bright's disease or other malady as disastrous in its results.

Dr. Larrabee: There is no more important subject for the consideration of the profession than this, and I must commend the presentation of Dr. Bond's ideas as he has expressed them. The prevalence of this disease is generally accepted to be on the increase. The fallacy of this belief may be explained by remembrance of the fact that they are better understood and more readily diagnosed now than formerly. It is doubtful whether the patient is in more danger from the disease than from the doctor who makes his diagnosis of the disease or eliminates it with the spirit-lamp and nitric acid. And we ought to do more in the particular line that Dr. Bond has indicated. No man supposes when he unbuttons his vest from distention induced by indigestion, or when he suffers from neuralgic pains, dimness of vision, etc., that he is on the outer borders of Bright's disease. Its frequency may be shown by instancing the fact that out of some 340 examinations of urine in a London hospital, half of the number, about 170, were found to have evidences of distinct lesions of the kidney.

Dr. J. M. Mathews, of Louisville, read a paper on "Obscure Rectal Diseases."

The term employed by Professor Goodell, "Hysterical Rectum," is, in my opinion, misleading, and while the importance of the affection cannot be overestimated, I am inclined to think a caption such as, "Some Obscure Affections of the Rectum," is better for the elucidation of the subject than that employed in Professor Goodell's paper, for the reason that it invites investigation. I dare say there is no physician here but has had some patient complain to him of some vague symptoms of rectal disease when such diagnosis was not borne out by an examination.

Dr. Goodell says, "In this form of hysteria there are usually present in my experience some one of the protean symptoms of general nerve prostration, such as spineaches and backaches, ovarian uneasiness, wakefulness, and nervousness. But the chief suffering or the most exacting symptom is referred to some portion of the rectal tract, leading the physician to suppose he is dealing with some case of traumatic lesion. The act of defecation then gives great suffering, followed by painful throbbing which may last for hours. Patients thus afflicted so dread the suffering that they school themselves into habits of costiveness, and often become victims to opium eating." But is Dr. Goodell correct when he says that one of these may lead the physician to suppose that he is dealing with some traumatic lesion? To state it more definitely, is not the physician really dealing with a traumatic lesion, and can a cure be effected upon any other hypothesis? Certain it is that if a lesion exists, the disease will not be cured until such lesion is eradicated.

He cited a number of cases to illustrate these conditions, and gave the following as causes of these obscure affections of the rectum.

1. Hysteria (?) neurosis.
2. The reflexes.
3. A lesion or pathological change at seat of trouble.

TREATMENT.

Taking it for granted that a pathological condition is found in the rectum, I shall only refer to those that would likely cause symptoms simulating the nervous rectum. (?) They are, viz.:

1. Fissure.
2. Ulcers.
3. Congestions.
4. Proctitis.
5. Internal fistula.

He then outlined the treatment of the different forms of rectal disease.

Dr. H. H. Mudd, of St. Louis, read a paper on "Contusions and Lacerations of the Kidney."

He gave in detail several cases, in one of which a boy aged five years had suffered a contusion, which, on the fifty-first day after injury, required nephrectomy. The operation was made through a lumbar incision, and the patient made a good recovery.

He called attention to the fact that in these injuries the peritoneum is not generally injured, and that a fluid tumor gradually developed in the lumbar region on the side injured.

A contusion without serious laceration of the kidney, but accompanied by a perinephritic hæmorrhage, might present all the diagnostic symptoms of a rupture. On the other hand, destructive laceration of the kidney-substance is slow in developing characteristic symptoms. Usually there is ample time for investigation of the cases before operative interference is demanded.

Dangerous conditions which may develop during the progress of such cases are shock, septicæmia, cystitis, with extension of inflammation through the bladder to the other kidney, and nephritis of the injured organ. Suppression of urine in both organs may occur during the presence of one or more of these conditions. Destructive unilateral nephritis may develop in the contused organ. Cuts, bullet wounds, and tears of kidney-substance heal quickly. Laceration in pelvis of the kidney or the ureter is much more likely to leave an intractable fistula. Incision, with inspection of the injured organ and drainage were recommended before extirpation. Laceration of the kidney may occur without external evidences of injury.

Dr. Wm. Townsend Porter gave "A Demonstration of the Effect of Cold, Applied to the Abdomen, Upon the Tracheal Circulation." It was made upon four cats, the trachea of each of which had been opened with a galvo-cautery knife. First, a warm poultice was applied to the belly of each cat, and on changing that for a cold application the change in circulation in the trachea was shown.

